# Environmental Risk Factors and Their Different Effects in Depressive Symptoms of Left-Behind Children in Rural China Compared with Non-Left-Behind Children

**DOI:** 10.3390/ijerph182010873

**Published:** 2021-10-16

**Authors:** Xiaoqing Zhang, Sharon A. Ray, Wei Hou, Xia Liu

**Affiliations:** 1 School of Health Technology and Management, Stony Brook University, New York, NY 11794, USA; xq.zhang@med.miami.edu; 2 Rehabilitation Science Department, University at Buffalo, New York, NY 14214, USA; sray5@buffalo.edu; 3 Department of Family, Population, and Preventive Medicine, Stony Brook University, New York, NY 11794, USA; wei.hou@stonybrookmedicine.edu; 4 Institute of Developmental Psychology, Faculty of Psychology, Beijing Normal University, Beijing 100875, China

**Keywords:** stressful life events, peer victimization, perceived discrimination, parental separation duration

## Abstract

In China, 61 million children were left behind in rural areas as a result of massive urbanization and migration of parents from the countryside to the cities in search of economic opportunities. This study explores the effects of environmental risk factors (i.e., peer victimization, perceived discrimination, and stressful life events) on depressive symptoms of left-behind children (LBC) and examines whether these risk factors have a higher impact on LBC compared with that of non-left-behind children (NLBC). Data collected involve 1548 first and second-year middle school students. Logistic regressions were conducted to examine the associations between environmental risk factors and LBC’s depressive symptoms, the moderating effect of the parental separation duration on these associations, and to compare if the risk factors had a higher impact on LBC’s depressive symptoms than on those of NLBC. Peer victimization is associated with an increased likelihood of depressive symptoms for LBC who were left behind for more than five years. Finally, stressful life events have a higher impact on LBC’s depressive symptoms, while peer victimization has a higher impact on NLBC’s depressive symptoms. The results suggest that stressful life events are an important risk factor that puts LBC at a disadvantage in terms of their mental health.

## 1. Introduction

The phenomenon of children left behind in rural China is a significant concern because of the reported negative mental health sequelae, including depression, anxiety, negative self-perception, and loneliness [1,2,3,4,5]. According to the All-China Women’s Federation (ACWF) [6], there is an estimate of 61 million left-behind children (LBC) whose parents have migrated to faraway urban cities for employment opportunities. LBC are left under the supervision of their grandparents, relatives, or elder-siblings, or are self-supervised. Parents rarely return home, and even when they do visit their children, they only stay for a very short periods of time. LBC were found to be at greater risk of suffering from depressive symptoms compared to non-left-behind children (NLBC) [7,8,9]. Being separated from their parents, LBC are at risk for poor attachment relationships between the child and the primary caregiver. LBC reported that they felt lonely, afraid, and even lost interest in life [10]. The depressive symptoms that LBC reported can represent a diagnosis of major depression and could negatively affect healthy growth and development, school performance, psychosocial functioning, and could potentially increase the likelihood of developing other psychiatric disorders in later life [11]. Therefore, a better understanding of what contributes to their depressive symptoms, especially in comparison with NLBC, is needed to inform policies and programs designed to address LBC’s mental health concerns.

### 1.1. Theoretical Background

As a public health framework, the ecological theory focuses both on the individual and the environment. The early advocate of ecological theory, Bronfenbrenner [12], claimed that children’s development is deeply affected by the family, school, peer, neighborhood, and community contexts in which they live. Within the ecological perspective, it is vital to identify and analyze risk factors at the level of the child (demographic risk factors) and his or her environment, which includes family, peer, school, and community (environmental risk factors), because both types of risk factors contribute to the child’s mental health outcomes. Previous studies characterized the demographic risk factors for LBC’s depression. For example, LBC from families with lower socioeconomic status (SES) are at a greater risk of developing depression than those with higher SES [13,14]. Family income is reported to be significantly associated with depressive symptoms of 1076 LBC from middle and high schools [15]. Being female is also found to be associated with more depressive symptoms [9,13]. However, from the ecological theory’s perspective, in addition to demographic risk factors, environmental risk factors should also be considered. An understanding of the child’s personal and environmental influences on mental health outcomes can help inform intervention design and delivery for these children.

### 1.2. Environmental Risk Factors and Depressive Symptoms

Existing studies showed that LBC report more peer victimization [16], perceived discrimination [17], and stressful life events [18]. Peer victimization is defined as experiencing maltreatment from peers, including physical, verbal, or relational aggression [19]. As LBC do not have their parents living with them for close supervision and care, they are vulnerable to abuse and violence. For example, Chen et al. (2017) surveyed 600 left behind middle school students and found that children left behind by their fathers experienced more peer victimization compared with that of children living with both parents in rural China [16]. As peer victimization was found to be associated with depressive symptoms of early adolescents [20,21], this study hypothesizes that peer victimization is positively correlated with LBC’s depressive symptoms.

The label of LBC is associated with perceived discrimination from other peers and society [17]. Discrimination occurs when someone is avoided or rejected due to stereotypes or prejudice; it refers to members of an ingroup taking a negative action against an outgroup or performing a positive action exclusively for the ingroup [22]. As a social stressor, discrimination can trigger a process of physiological responses (e.g., elevated blood pressure and heart rate); these heightened physiological responses over time can have downstream effects on health [23]. LBC consistently reported perceived discrimination by their peers or teachers [17,24], and this perceived discrimination was found to be a strong predictor of negative mental health outcomes [24,25,26]. This study hypothesizes that perceived discrimination would be positively correlated with LBC’s depressive symptoms.

In the literature, LBC are reported to experience more stressful life events than NLBC with reported poorer mental health outcomes [18,27,28,29]. Stressful life events can include: interpersonal conflict (i.e., conflicts between self and others), academic stress (i.e., failures in exam or heavy course load), punishment (i.e., criticism or corporal punishment), loss (i.e., death of a loved one or loss of property), health and adaptation problems (i.e., severe illnesses and maladjustment to changed diet, daily routine or living environments), et cetera [30]. There was no study that explored these environmental risk factors with one model with their predicting effects on LBC’s depressive symptoms. Furthermore, previous studies did not use non-left-behind children (NLBC) as a comparison group or examine whether environmental risk factors have differential effects on predicting LBC and NLBC’s depressive symptoms. Identifying risk factors in one model can help to prioritize what to focus on when designing intervention programs aimed at improving LBC’s psychological health.

Therefore, it is hypothesized that environmental risk factors are positively correlated with LBC’s depressive symptoms, controlling for the demographic risk factors (i.e., gender, family income, perceived SES, and parents’ education). This study also examines which risk factors have a higher impact on depressive symptoms in LBC than NLBC. Additionally, research findings suggested that the longer the children were left behind, the more depression symptoms they exhibited [31,32]. This study hypothesizes that environmental risk factors have a higher impact on the depressive symptoms of children who were separated from their parents for a longer time. 

## 2. Materials and Methods

### 2.1. Participants and Procedure

In 2013, Henan province reported having the second largest population of LBC, composed of 10.73% of the whole population of LBC in China [6]. The first- and second-year middle school students from Henan province participated in a cross-sectional survey. A total of 1572 students filled out the survey. They ranged in age from 11–15 years. The survey was conducted in October 2018. With the facilitation of the teachers and school administrators, students who agreed to complete the questionnaire were given consent and assent forms and were debriefed on the nature and purpose of the study. After the consent and assent processes were completed, students completed the questionnaire in the classrooms overseen by the research team in China and provided demographics, including gender, age, grade, family monthly income, and parental education levels. Participants also reported their perceived socioeconomic status (SES), which was measured by the rung that the respondents chose to represent their social classes (01 being very low socioeconomic status to 10 being very high) [33]. The response rate in the classroom survey was 100%. The study was approved by the Human Research Ethics Committee of Beijing Normal University. The current research using the de-identified dataset was approved by the Institutional Review Board of Stony Brook University. Below are the measurements included in this questionnaire. 

### 2.2. Outcome Measures

Children’s depressive symptoms were measured with the Center for Epidemiological Studies Depression Scale for Children (CES-DC), a 20-item four-point self-report Likert scale (0 = ‘not at all’ to 3 = ‘a lot’). Respondents reported how often they experienced the symptom described in each question during the past week. Using CES-DC, children with a score of depression more than 15 were categorized as those with depressive symptoms [34]. Children with a score of 15 or less were categorized as those with no depressive symptoms. This study uses the average scores. Therefore, the threshold score for depressive status is 0.75.

### 2.3. Comparison Group

Children were considered as ‘left-behind’ when they indicated in the survey that one or both of their parents had migrated to urban cities to work. They also reported how long it was since their parents began to migrate for work as an indicator of the parental separation duration. Those children who had no history of being left behind and lived with both parents at their original residences were considered NLBC.

### 2.4. Risk Factors

Peer victimization. An adapted version of the Multidimensional Peer-Victimization Scale [19] was used to measure four types of peer victimization: physical victimization, verbal victimization, social manipulation, and attacks on property. This scale consists of 21 items rated on a four-point Likert scale (0 = ‘never happened’ to 3 = ‘always happened’).

Perceived discrimination. The modified version of the perceived discrimination survey [35] has two constructs consisting of personal perceived discrimination (e.g., I feel that I am treated unfairly) and group perceived discrimination (e.g., kids with migrated parents like me are being looked down upon). The survey is a six-item, five-point Likert scale (1 = ‘not true at all’ to 5 = ‘totally true’).

Stressful life events. Stressful life events were measured with the Adolescent Self-Rating Life Events Checklist (ASLEC) [30], which includes 27 items falling into 6 dimensions that are referred to as interpersonal conflict, academic stress, punishment, loss, health and adaptation problems, and all other types of events. Participants answered how badly the listed life events had impacted them. The checklist is a six-point Likert scale (1 = ‘never happened’, 2 = ‘no impact’, 3 = ‘slightly’ to 6 = ‘extremely badly’).

### 2.5. Data Analysis

Chi-square tests were used to compare the percentages of gender, father’s education, mother’s education, family income, and perceived SES between LBC and NLBC. Analysis of covariance was conducted to determine whether there were any statistically significant differences in CES-DC score between LBC and NLBC with adjustment for gender, family income, perceived SES, and parents’ education. 

To evaluate the associations between risk factors and depressive symptoms and the moderating effect of the parental separation duration on these associations, logistic regression models were performed with the dependent variable being whether the participant has depressive symptoms or not (CES-DC > 0.75 is considered depressive status). Peer victimization, perceived discrimination, and stressful life events were included as primary independent variables for the first model. Parental separation duration and three interaction terms (created using parental separation duration multiplied by the three risk factors, respectively) were added as independent variables for the second model. Gender, family income, perceived SES, and parents’ education were adjusted as covariates. To examine whether risk factors had a higher impact on depressive symptoms in LBC than NLBC, another logistic regression was performed incorporating LBC/NLBC status and three interaction terms created using LBC/NLBC status multiplied by three risk factors, respectively. Spearman’s correlations among peer victimization, perceived discrimination, and stressful life events were used to check their multicollinearity. The linearity of the variables with respect to the logit of the dependent variable was assessed via the Box-Tidwell (1962) procedure [36]. Outliers were tested using a Casewise List table generated by the binomial logistic regression. All analyses were conducted using IBM SPSS software version 26 (IBM, Armonk, NY, USA).

## 3. Results

### 3.1. Demographic Information

The final study sample includes 1548 participants, with 841 LBC (54.3 percent), 707 NLBC (45.7 percent) after excluding 24 participants who did not indicate their parental migration status (Table 1). There were 47 percent males for the LBC group and 55.9 percent males for the NLBC group. The mean age was 12.71 for LBC and 12.63 for NLBC (*p* < 0.0005). Mother’s education and family income differed by left-behind status (both *p* < 0.0005). The observed frequency and percentage of each category for each variable for both LBC and NLBC groups were presented in Table 1. CES-DC, peer victimization, perceived discrimination, and stressful life events all demonstrate high internal consistency (Cronbach’s alpha ranged from 0.76 to 0.89).

### 3.2. CES-DC Depressive Symptom Scores

The results of the analysis of covariance indicated that LBC (mean = 0.93, SD = 0.49) had a significantly higher level of depressive symptoms than NLBC (mean = 0.86, SD = 0.48) with adjustment for gender, family income, perceived SES, and parents’ education (F = 5.85, *p* = 0.016), which is consistent with the previous research findings. 

### 3.3. Correlations of Risk Factors on LBC’s Depressive Symptoms

For the first model, all three predictor variables were statistically significant in the multiple logistic regression model (all *p* < 0.05) (Table 2). For each unit increase in peer victimization, perceived discrimination, and stressful life events, the odds of having depressive symptoms increased by a multiplicative factor of 1.86 (95 percent CI = 1.06–3.28, *p* = 0.032), 1.30 (95 percent CI = 1.02–1.66, *p* = 0.036), and 5.85 (95 percent CI = 3.85–8.90, *p* < 0.0005), respectively. The odds of having depressive symptoms increased by a multiplicative factor of 1.51 if the child was female (OR = 1.51, 95 percent CI = 1.07–2.15, *p* = 0.021).

**Figure 1:**
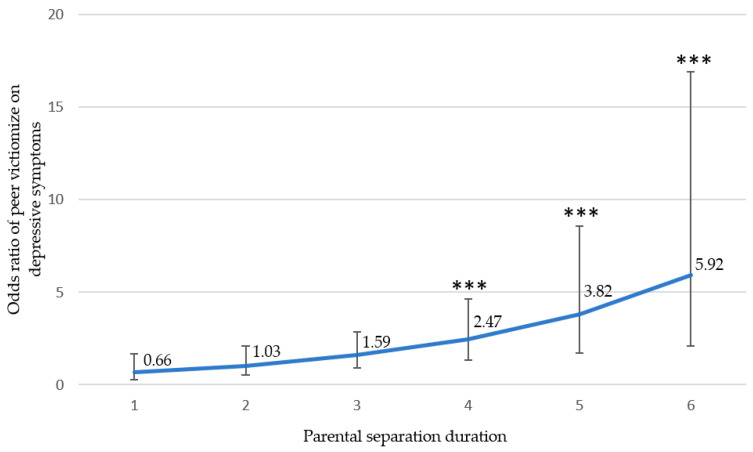
Odds ratios of peer victimization on depressive symptoms for different parental separation duration. Notes: *** indicates *p* < 0.001 for a two-tailed test. 1 = less than half a year, 2 = half a year to 1 year, 3 = 2 to 4 years, 4 = 5 to 7 years, 5 = 8 to 10 years, and 6 = more than 10 years.

For the second model where we examined how parental separation duration moderated the associations between risk factors and LBC’s depressive symptoms, the results indicated a significant interaction effect between parental separation duration and peer victimization on depressive symptoms (*p* = 0.006, Table 3). To illustrate the moderating effects of parental separation duration, the odds ratios of peer victimization on depressive symptoms were calculated at each time period: less than half a year, half a year to 1 year, 2 to 4 years, 5 to 7 years, 8 to 10 years, and more than 10 years. The odds ratios of peer victimization for each time period were 0.66, 1.03, 1.59, 2.47, 3.82, and 5.92, respectively (Figure 1). The *p* values of the last three separation durations (5 to 7 years, 8 to 10 years, and more than 10 years) were significant (all *p* < 0.001), suggesting that peer victimization was associated with an increased likelihood of depressive symptoms for LBC who had been left behind for more than five years.

### 3.4. Difference in the Correlations of Risk Factors on Depressive Symptoms between LBC and NLBC

When we incorporated three risk factors and three corresponding interaction terms created using LBC/NLBC status multiplied by three risk factors, no significant interaction effect was found between LBC group, perceived discrimination, and depressive symptoms. Therefore, perceived discrimination * LBC was removed from the logistic regression. Based on the final model, both interaction terms (peer victimization * LBC and stressful life events * LBC) were significant (*p* = 0.025 and *p* = 0.046, respectively) (Table 4). The odds ratios of peer victimization for both LBC and NLBC were calculated, and they were 2.13 for LBC and 5.55 for NLBC. Similarly, the odds ratios of stressful life events for LBC and NLBC were 6.51 and 3.65 (Table 5). For each unit increase in peer victimization, the odds of having depressive symptoms increased by a multiplicative factor of 2.13 for LBC and 5.55 for NLBC; for each unit increase in stressful life events, the odds of having depressive symptoms increased by a multiplicative factor of 6.51 for LBC and 3.65 for NLBC. Therefore, stressful life events had a significantly higher impact on LBC than NLBC.

## 4. Discussion

This is the first study that discusses the contributions to LBC’s depressive symptoms by incorporating three important environmental risk factors and controlling for the demographic risk factors. This study further examines which environmental risk factors have a higher impact on the depressive symptoms of LBC than NLBC. The importance of this research is threefold: (1) it applies the ecological theory to utilize a more holistic model by examining risk factors at the environmental level while controlling for those at the individual level, and to explain the challenges that LBC face that are linked to an increased likelihood of developing depressive symptoms; (2) it compares the different impacts of the environmental risk factors on LBC and NLBC’s depressive symptoms, showing which risk factors may have a greater impact on LBC, and suggests where to prioritize when designing intervention programs aimed at improving LBC’s psychological health; and (3) it can suggest from the intervention perspective which environmental risk factors should be further explored as protective factors to protect LBC from developing depressive symptoms when encountering those risk factors. Understanding how to address these potential risk and protective factors can inform policies that reduce the LBC’s likelihood of developing depressive symptoms.

In this study, LBC have a significantly higher level of depressive symptoms than NLBC, which is consistent with the previous research findings [7,13,37]. Stressful life events have the highest impact on LBC’s depressive symptoms compared with peer victimization and perceived discrimination, and it has a higher impact on the depressive symptoms of LBC than NLBC. This provides statistical evidence that stressful life events are an important factor that puts LBC at a disadvantage compared with their NLBC counterparts. The loss of parents in the home for care and guidance can exacerbate the effects of stressful life events on LBC, including stress from schoolwork, relationship with friends, family-related issues, and self-care, as measured in the questionnaire [18]. Therefore, further research examining what protective factors protect LBC from the negative impact of stressful life events on their mental health outcome will be meaningful for informing effective intervention programs.

By contrast, daily stressful life events may not have a significantly higher impact on NLBC as their negative impact might be mitigated by their parents being present at home. Nevertheless, more serious stressors such as peer victimization have a negative impact on both LBC and NLBC, and they have a higher impact on NLBC. Peer victimization refers to dysfunctional peer relationships. LBC can be mocked by their peers for being abandoned by their parents. They can easily be the targeted victims of bullying if their peers believe that they don’t have parents to support them. However, peer victimization may not necessarily have a higher impact on LBC than NLBC. On the one hand, peer victimization is very prevalent among adolescents, especially in the context of school [38]; on the other hand, because of knowing that parents of LBC are not present at home, the local government and schools put supports in place to keep LBC safe from serious negative events such as peer victimization. Along the same line, in 2016, the National Health and Family Planning Commission of China (NHFPC) released an announcement about improving care for LBC’s well-being with an emphasis on requiring that schools report to the government any negative incidents that happen to LBC [39]. Therefore, peer victimization may not be the main concern for LBC.

In addition, parental separation duration moderates the positive association between peer victimization and LBC’s depressive symptoms. In particular, peer victimization has a higher impact on the depressive symptoms of children who are left behind for longer periods. This suggests that when designing intervention programs to reduce the incidents of peer victimization and its effect on depressive symptoms, special attention should be given to LBC who experience a longer period of separation from their parents. 

Perceived discrimination is a significant risk factor for LBC’s depressive symptoms. Perceived discrimination against LBC occurs mostly when those children are excluded or rejected by their peers [40,41]. As the LBC phenomenon attracts more and more attention from society with increasing news reports on their antisocial life attitude, depression, and behavioral problems, they are stigmatized and labeled as problematic children [35]. This may lead to peer rejection and discrimination against LBC. Future intervention design can consider educating local communities to be more inclusive and supportive for LBC. Additionally, parenting training that guides migrant parents on how to maintain effective communication and stay connected with their children can potentially prevent LBC from the negative impact of perceived discrimination [41]. 

### Limitations

Despite the contributions to the literature, the present study has certain limitations that are worth noting. First of all, this study is a cross-sectional study that cannot determine if the risk factors included in the study have similar effects on LBC’s depressive symptoms over time. Longitudinal studies using data from multiple time points would be necessary to show robust effects of the risk factors on LBC’s depressive symptoms. Moreover, this research utilizes self-report survey results, which may have potential validity problems. Participants may exaggerate symptoms or under-report their feelings. They may also simply misunderstand or misremember the questions covered in the survey. Gathering information from various sources such as LBC’s parents, teachers, and peers could address these potential validity issues.

## 5. Conclusions

Controlling for demographic risk factors, environmental risk factors including peer victimization, perceived discrimination, and stressful life events are significantly associated with the likelihood for developing depressive symptoms in left-behind children (LBC). Peer victimization is associated with an increased likelihood of depressive symptoms for LBC who were left behind for more than five years. More importantly, in addition to having the largest effect on LBC’s depressive symptoms compared with that of peer victimization and perceived discrimination, stressful life events exhibit a higher impact on LBC’s depressive symptoms than non-left-behind children (NLBC), putting LBC at a disadvantage in terms of their mental health. Therefore, the priority is to reduce the stressful life events that happened to LBC and improve their ability to cope with such events. In general, to protect the LBC from exhibiting depressive symptoms, future research can either explore how to reduce peer victimization, perceive discrimination, and stressful life events, or identify and examine which protective factors can mitigate the negative effects of those risk factors. 

## Figures and Tables

**Table 1:** Chi-square analysis on characteristics by parental migration status.

		LBC Total N = 824 N (%)	NLBC Total N = 698 N (%)	*p* Value
Age (Mean, SD)		12.71 (0.796)	12.63 (0.833)	0.047
Gender				0.648
	Male	470 (57.0%)	390 (55.9%)	
	Female	354 (43.0%)	308 (44.1%)	
Father’s education				0.071
	1 = primary school	155 (18.7%)	117 (16.9%)	
	2 = middle school	441 (53.3%)	346 (50.0%)	
	3 = high school or technical secondary school	181 (21.9%)	163 (23.6%)	
	4 = junior college	25 (3.0%)	38 (5.5%)	
	5 = college and above	25 (3.0%)	28 (4.0%)	
Mother’s education				<0.0005
	1 = primary school	240 (29.4%)	170 (24.5%)	
	2 = middle school	430 (52.6%)	336 (48.4%)	
	3 = high school or technical secondary school	115 (14.1%)	135 (19.5%)	
	4 = junior college	17 (2.1%)	26 (3.7%)	
	5 = college and above	15 (1.8%)	27 (3.9%)	
Family income/month (USD)				<0.0005
	1 = less than 72.57	4 (0.5%)	6 (0.9%)	
	2 = 72.57–145.14	24 (3.1%)	25 (3.7%)	
	3 = 145.14–290.28	79 (10.1%)	68 (10.2%)	
	4 = 290.28–580.55	194 (24.7%)	203 (30.4%)	
	5 = 580.55–870.83	172 (21.9%)	181 (27.1%)	
	6 = 870.83–1161.10	175 (22.3%)	115 (17.2%)	
	7 = more than 1161.10	137 (17.5%)	69 (10.3%)	
Perceived SES				0.135
	1	9 (1.1%)	1(0.1%)	
	2	16 (1.9%)	7 (1.0%)	
	3	72 (8.7%)	52 (7.5%)	
	4	151 (18.2%)	116 (16.7%)	
	5	308 (37.2%)	264 (38.0%)	
	6	163 (19.7%)	132 (19.0%)	
	7	68 (8.2%)	73 (10.5%)	
	8	31 (3.7%)	38 (5.5%)	
	9	6 (0.7%)	7 (1.0%)	
	10	4 (0.5%)	4 (0.6%)	

Notes: Age difference is based on *t*-test. CNY-USD currency exchange rate is 1 Chinese Yuan equals 0.15 U.S. Dollar, 15 January 2020. Abbreviations: LBC, left-behind children; NLBC, non-left-behind children; SD, standard deviation; SES, socioeconomic status.

**Table 2:** Logistic regression estimates (risk factors) of depressive symptoms among LBC.

	B	SE	OR	95% CI for OR		*p* Value
				Lower	Upper	
Independent variables						
Peer victimization	0.62 *	0.29	1.86	1.06	3.28	0.032
Perceived discrimination	0.26 *	0.13	1.30	1.02	1.66	0.036
Stressful life events	1.77 ***	0.21	5.86	3.86	8.90	0.000
Female	0.41 *	0.18	1.51	1.07	2.15	0.021
Family income	–0.03	0.07	0.97	0.86	1.11	0.692
Father’s education	–0.07	0.12	0.93	0.74	1.17	0.535
Mother’s education	0.09	0.12	1.10	0.86	1.39	0.452
Perceived SES	–0.12	0.07	0.89	0.78	1.02	0.092
Intercept	−4.07 ***	0.68				0.000
χ^2^	205.12 ***					
Nagelkerke R^2^	0.33					
Observations	740					
Highest VIF	1.45					
Mean VIF	1.38					

Notes: * indicates *p* < 0.05, *** *p* < 0.001 for a two-tailed test. Abbreviations: LBC, left-behind children; B, unstandardized beta; SE, standard error; OR, odds ratio; CI, confidence interval; SES, socioeconomic status; VIF, variance inflation factor.

**Table 3:** Logistic regression estimates of risk factors and parental separation duration on depressive symptoms among left-behind children (LBC).

	B	SE	OR	95% CI for OR		*p* Value
				Lower	Upper	
Independent variables						
PV	0.75 *	0.31	2.11	1.16	3.83	0.014
PD	0.33 *	0.13	1.39	1.08	1.79	0.011
SLE	1.72 ***	0.22	5.57	3.63	8.55	0.000
PSD	0.025	0.06	1.03	0.92	1.14	0.65
PV * PSD	0.44 **	0.16	1.55	1.13	2.12	0.006
PD * PSD	0.04	0.07	1.04	0.90	1.19	0.63
SLE * PSD	–0.01	0.12	0.99	0.78	1.26	0.93
Female	0.40 *	0.18	1.49	1.04	2.14	0.031
Family income	–0.05	0.07	0.96	0.83	1.09	0.51
Father’s education	–0.03	0.12	0.97	0.77	1.22	0.77
Mother’s education	0.07	0.12	1.07	0.84	1.37	0.58
Perceived SES	–0.13	0.07	0.88	0.77	1.02	0.08
Intercept	1.21 *	0.47				0.01
χ^2^	208.18 ***					
Nagelkerke R^2^	0.342					
Observations	712					

Notes: * indicates *p* < 0.05, ** *p* < 0.01, *** *p* < 0.001 for a two-tailed test. Abbreviations: LBC, left-behind children; B, unstandardized beta; SE, standard error; OR, odds ratio; CI, confidence interval; PV, peer victimization; PD, perceived discrimination; SLE, stressful life events; PSD, parental separation duration; SES, socioeconomic status.

**Table 4:** Logistic regression estimates of risk factors and left-behind status on depressive symptoms.

	B	S.E.	OR	95% CI for OR		*p* Value
				Lower	Upper	
Independent variables						
Peer victimization	1.71 ***	0.324	5.55	2.94	10.46	0.000
Stressful life events	1.29 ***	0.20	3.65	2.46	5.41	0.000
LBC	0.28 *	0.13	1.32	1.01	1.71	0.039
Peer victimization * LBC	–0.96 *	0.43	0.38	0.17	0.89	0.025
Stressful life events * LBC	0.58 *	0.29	1.78	1.01	3.15	0.046
Female	0.47 ***	0.13	1.60	1.24	2.06	0.000
Family income	–0.04	0.05	0.96	0.87	1.06	0.426
Father’s education	–0.01	0.08	1.00	0.85	1.17	0.954
Mother’s education	0.04	0.08	1.04	0.88	1.23	0.636
Perceived SES	–0.09	0.05	0.91	0.83	1.01	0.066
Intercept	0.68 *	0.33				0.037
χ^2^	368.56 ***					
Nagelkerke R^2^	0.32					
Observations	1370					

Notes: * indicates *p* < 0.05, *** *p* < 0.001 for a two-tailed test. LBC is a categorical variable, with 1 being left-behind children and 0 being non-left-behind children. Abbreviations: B, unstandardized beta; SE, standard error; OR, odds ratio; CI, confidence interval; LBC, left-behind children; SES, socioeconomic status.

**Table 5:** Odds ratios of risk factors on depressive symptoms for LBC and NLBC.

		OR	95% CI for OR		*p* Value
			Lower	Upper	
Peer victimization	LBC	2.13	1.22	3.70	0.0007
	NLBC	5.55	2.94	10.46	<0.0001
Stressful life events	LBC	6.51	4.30	9.84	<0.0001
	NLBC	3.65	2.46	5.41	<0.0001

Notes: N (LBC) = 740, N (NLBC) = 626. Abbreviations: LBC, left-behind children; NLBC, non-left-behind children; OR, odds ratio; CI, confidence interval.

## Data Availability

The data presented in this study are openly available in Mendeley Data at DOI: 10.17632/pjwzhw8s2x.1.

## References

[1] Fan F., Su L., Gill M.K., Birmaher B. (2010). Emotional and behavioral problems of Chinese left-behind children: A preliminary study. Soc. Psychiatry Psychiatr. Epidemiol..

[2] Gao Y., Li L., Kim J.H., Congdon N., Lau J., Griffiths S. (2010). The impact of parental migration on health status and health behaviors among left behind adolescent school children in China. BMC Public Health.

[3] Guo J., Chen L., Wang X., Liu Y., Chui C.H.K., He H., Qu Z., Tian D. (2012). The relationship between Internet addiction and depression among migrant children and left-behind children in China. Cyberpsychol. Behav. Soc. Netw..

[4] Sun X., Tian Y., Zhang Y., Xie X., Heath M.A., Zhou Z. (2015). Psychological development and educational problems of left-behind children in rural China. Sch. Psychol. Int..

[5] Wen M., Lin D. (2012). Child development in rural China: Children left behind by their migrant parents and children of nonmigrant families. Child Dev..

[6] All-China Women’s Federation (ACWF, 2013) Report on the Status Quo of Left-Behind Children in Rural China. https://wenku.baidu.com/view/a5011585192e45361066f5cb.html.

[7] He B., Fan J., Liu N., Li H., Wang Y., Williams J., Wong K. (2012). Depression risk of “left-behind children” in rural China. Psychiatry Res..

[8] Wang Y., Xiao L., Rao W., Chai J., Zhang S., Ng C.H., Ungvari G.S., Zhu H., Xiang Y. (2019). The prevalence of depressive symptoms in “left-behind children” in China: A meta-analysis of comparative studies and epidemiological surveys. J. Affect. Disord..

[9] Wu Q., Lu D., Kang M. (2015). Social capital and the mental health of children in rural China with different experiences of parental migration. Soc. Sci. Med..

[10] Ye J., Pan L. (2011). Differentiated childhoods: Impacts of rural labor migration on left-behind children in China. J. Peasant Stud..

[11] Rey J.M., Bella-Awusah T.T., Liu J. (2015). Depression in Children and Adolescents. in IACAPAP Textbook of Child and Adolescent Mental Health. Lancet.

[12] Bronfenbrenner U. (1979). The Ecology of Human Development: Experiments by Nature and Design.

[13] Liu Z., Li X., Ge X. (2009). Left too early: The effects of age at separation from parents on Chinese rural children’s symptoms of anxiety and depression. Am. J. Public Health.

[14] Yang Y., Tao F., Wan Y. (2010). Depressive symptoms and the influencing factors among left-behind children. Chin. J. Sch. Health.

[15] Tan M., Chen M., Li J., He X., Jiang Z., Tan H., Huang X. (2018). Depressive symptoms and associated factors among left-behind children in China: A cross-sectional study. BMC Public Health.

[16] Chen X., Liang N., Ostertag S.F. (2017). Victimization of children left behind in rural China. J. Res. Crime Delinq..

[17] Zhang L., Fu W., Wang D., Bao Z. (2015). The Discrimination perception and problem behaviors of children left-behind in the middle school—A qualitative study. Chin. J. Spec. Educ..

[18] Liu B., Wang W. (2010). Study on living stress events and psychological health of children remaining in rural areas. China J. Health Psychol..

[19] Mynard H., Joseph S. (2000). Development of the multidimensional peer-victimization scale. Aggress. Behav. Off. J. Int. Soc. Res. Aggress..

[20] Hawker D.S., Boulton M.J. (2000). Twenty years’ research on peer victimization and psychosocial maladjustment: A meta-analytic review of cross-sectional studies. J. Child Psychol. Psychiatry Allied Discip..

[21] Zhao J., Yang P., Ma J., Huang C. (2016). Perceived discrimination and positive/negative emotion of left-behind children: The protective role of parent-child cohesion. Psychol. Dev. Educ..

[22] Rosenberg J., Rosenberg S.J. (2013). Community Mental Health: Challenges for the 21st Century.

[23] Pascoe E.A., Smart Richman L. (2009). Perceived discrimination and health: A meta-analytic review. Psychol. Bull..

[24] Zhang L. (2011). A study of social relationships and loneliness of the rural left-behind children. Chin. J. Clin. Psychol..

[25] Fan L., Shao J. (2013). The family economic status of children left behind and their subjective well-being: Mediating role of perceived discrimination. J. Guizhou Norm. Univ. Nat. Sci..

[26] Zhao J., Yang P., Zhao X., Zhang W. (2016). Early adolescence’s peer victimization and depression: The moderating effects of self-esteem and gender. Chin. J. Spec. Educ..

[27] Guang Y., Feng Z., Yang G., Yang Y., Wang L., Dai Q., Hu C., Liu K., Zhang R., Xia F. (2017). Depressive symptoms and negative life events: What psycho-social factors protect or harm left-behind children in China?. BMC Psychiatry.

[28] Han L., Zhao S., Pan X., Liao C. (2018). The impact of students with left-behind experiences on childhood: The relationship between negative life events and depression among college students in China. Int. J. Soc. Psychiatry.

[29] Hu X., Liu X., Shen J., Fan X. (2007). The affection of life events and coping styles on left children’s mental health. Chin. J. Clin. Psychol..

[30] Liu X., Liu L., Yang J., Cai F., Wang A., Sun L., Zhao G., Ma D. (1997). The adolescent self-rating life events checklist and its reliability and validity. Chin. J. Clin. Psychol..

[31] Ling H., Zhang J.R., Yi Y., Zhou L.J., Hong W.Y., Wen J. (2012). Impact of the age and duration separated from parents on left-home-kids’ behaviors and emotions. Chin. J. Clin. Psychol..

[32] Zhang L., Luo X.R., Meng R.H. (2010). Status quo of emotion problems of left-behind children in rural areas of Changzhi City. Chin. J. Health Educ..

[33] Goodman E., Adler N.E., Kawachi I., Frazier A.L., Huang B., Colditz G.A. (2001). Adolescents’ perceptions of social status: Development and evaluation of a new indicator. Pediatrics.

[34] Weissman M.M., Orvaschel H., Padian N. (1980). Children’s symptom and social functioning self-report scales: Comparison of mothers’ and children’s reports. J. Nerv. Ment. Disord..

[35] Shen J., Hu X., Liu X. (2009). Left-over children’s perceived discrimination: Its characteristics and relationship with personal well being. J. Henan Univ. Soc. Sci..

[36] Box G.E.P., Tidwell P.W. (1962). Transformation of the independent variables. Technometrics.

[37] Liang Y., Wang L., Rui G. (2017). Depression among left-behind children in China. J. Health Psychol..

[38] Liu X., Lu D., Zhou L., Su L. (2013). Relationship between bullying, victimization and depression, suicidal ideation. Chin. J. Clin. Psychol..

[39] National Health and Family Planning Commission of China (NHFPC, 2016) Annoucement on How to Work on Caring for Left-Behind Children’s Well-Being. http://www.nhc.gov.cn/ldrks/s7846/201605/db26bb1b7f9140b8b4b8c2e019edca69.shtml.

[40] Chen H., Liu Q., Hu B. (2011). Study on the social support and peer relationships impact on psychological resilience among the left-behind children in rural area. Mod. Prev. Med..

[41] Zhao J., Liu X., Zhang W. (2013). Peer rejection, peer acceptance and psychological adjustment of left-behind children: The roles of parental cohesion and children’s cultural beliefs about adversity. Acta Psychol. Sin..

